# Endophytic Fungal Volatile Compounds as Solution for Sustainable Agriculture

**DOI:** 10.3390/molecules24061065

**Published:** 2019-03-18

**Authors:** Amine Kaddes, Marie-Laure Fauconnier, Khaled Sassi, Bouzid Nasraoui, Mohamed-Haïssam Jijakli

**Affiliations:** 1 Urban and Integrated Plant Pathology Laboratory, Gembloux Agro-Bio Tech (GxABT), University of Liège, 5030 Gembloux, Belgium; mh.jijakli@uliege.be; 2 General and Organic Chemistry Unit, Gembloux Agro-Bio Tech (GxABT), University of Liège, 5030 Gembloux, Belgium; marie-laure.fauconnier@uliege.be; 3 Department of Agronomy and Plant Biotechnology, National Agronomic Institute of Tunisia, University of Carthage, Tunis 1082, Tunisia; khaledsassi1@gmail.com; 4 RL/Biogressors and Integrated Protection in Agriculture, National Agronomic Institute of Tunisia, University of Carthage, Tunis 1082, Tunisia; nasraouibouzid2012@gmail.com

**Keywords:** volatile organic compounds, fungi, endophytic fungi, pathogenic

## Abstract

Endophytic fungi produce various mixtures of carbon-based compounds, which are known as volatile organic compounds (VOCs). Research regarding the use of VOCs as pesticide substitutes has garnered much attention. This review summarizes the recent knowledge about VOCs regarding their origin and chemical properties and emphasizes their antimicrobial potential against a wide variety of agricultural pathogens. Several studies have highlighted the importance of VOCs as antimicrobial agents. Nevertheless, the application of VOCs in biofumigation methods still requires the advanced evaluation of their phytotoxicity.

## 1. Introduction

The control of pre- and post-harvest diseases is essential to maintain the quality of crops and agricultural products. Currently, while the application of pesticides is the most popular way to control several pathogens, there is irrefutable evidence that the use of these products is harmful to humans and the environment, besides causing the proliferation of pathogen-resistant strains [1,2]. In this context, the development of a biopesticide strategy, as an alternative to conventional pesticides, has become a research topic of great interest. Biocontrol methods are based on the use of either living organisms or natural substances produced by these organisms (pheromones, plant extract) [3]. The exploitation of natural substances, such as essential oils, seems to be a promising approach for controlling post-harvest diseases caused by different micro-organisms, producing safe foods, and reducing environmental pollution. Moreover, natural substances could effectively control the growth of certain pesticide-resistant microorganisms [4]. In the context of biocontrol methods, it has been reported that several endophytic fungi are able to produce volatile organic compounds (VOCs) [5,6]. Endophytic fungi are defined as fungi that spend the whole or part of their lifecycle colonizing inter-and/or intra-cellular systems, especially leaves, stems, and roots, without causing any apparent disease symptoms in their hosts [7,8]. Endophytic fungi play important roles in plant micro-ecosystems. Reports have revealed that over 1 million fungal endophytes have been found in various plant micro-ecosystems [9]. Endophytic fungi provide many benefits to their hosts by enhancing host growth and defense against pathogens. In addition, many endophytic fungal strains, such as *Trichoderma*, *Noduliosporium*, and *Muscodor* sp., can modulate the plant defense systems by inhibiting and reducing the load of fungal pathogens [10]. These benefits are principally attributed to the mixture of VOCs produced by endophytic fungi. In fact, there is an estimated 322 identified secondary metabolites secreted by endophytic fungi. These metabolites are derived from different fungal metabolism pathways and consist of a diverse range of compounds, including alkaloids, terpenoids, quinones, peptides, xanthones, and phenols [11,12]. Owing to their small size and high vapor pressure, VOCs can diffuse easily through the atmosphere and soil [13]. It is known that these compounds play an important role in communication between fungi and other organisms in the ecosystem. In addition, these molecules have shown promising bioactivity potential against a wide range of pathogens [14,15]. Hence, these molecules are used as part of biocontrol strategies, in what is termed mycofumigation, to inhibit the growth of numerous plant pathogens [16,17].

As a single review cannot discuss the findings of the different studies on fungal VOCs, this review focuses only on endophytic fungal VOCs, with an emphasis on their structure, properties, production, and biological activities.

## 2. Volatile Organic Compounds (VOCs)

VOCs are hydrophobic, organic molecules with a low molecular weight (<300 Da) and high vapor pressure (≥0.01 kPa at 20 °C) [18]. Due to their physico-chemical properties, these molecules can cross plant cell membranes; therefore, they play a very important role in the functioning of soil ecosystems [13,19,20]. VOCs are of anthropic (refining, evaporation of organic solvents, unburned, etc.,) or natural origins (emissions by plants, animals, and microorganisms). The majority belong to five chemical groups—terpenoids, fatty acid derivatives, benzenoid compounds, phenylpropanoids, and amino acid derivatives. They play a very important role in the control of several fungal pathogens [20,21].

**Figure 1:**
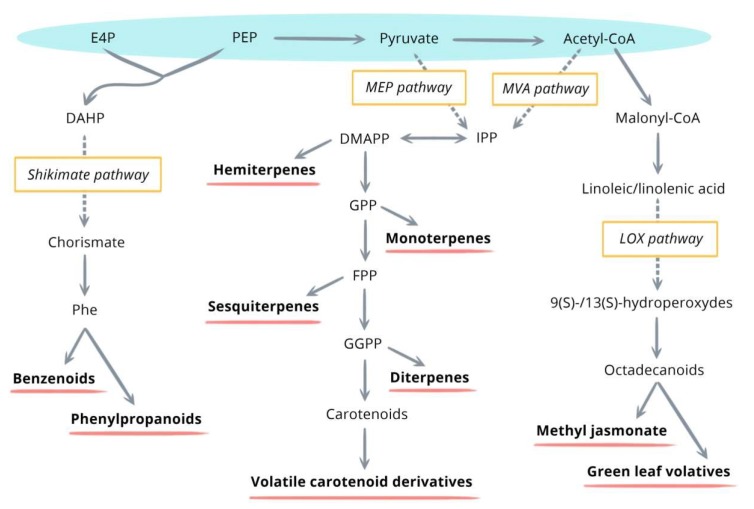
General overview of the major metabolic pathways inducing the synthesis of volatile organic compounds (VOCs.) [20]. DAHP, 3-deoxy-D-arabinoheptulosonate-7 phosphate; DMAPP, dimethylallyl pyrophosphate; E4P, erythrose 4-phosphate; FPP, farnesyl pyrophosphate; GGPP, geranylgeranyl pyrophosphate; GPP, geranyl pyrophosphate; IPP, isopentenyl pyrophosphate; NPP, neryl pyrophosphate; PEP, phosphoenolpyruvate; Phe, phenylalanine.

Their biosynthesis is highly dependent on the availability of carbon, nitrogen, sulfur, and energy provided by primary metabolism. Four precursors are known to be implicated in these biosynthetic pathways—PEP, E4P, pyruvate, and acetyl-coA. The four major metabolic pathways, namely the shikimate/phenylalanine, the mevalonic acid (MVA), the methylerythritol phosphate (MEP), and lipoxygenase (LOX) pathways, involve different enzymatic reactions and lead to the production of benzenoids/phenylpropanoids, sesquiterpenes, monoterpenes, hemiterpenes, diterpenes, volatile carotenoid derivatives, and methyl jasmonate/green leaf volatiles (Figure 1) [20].

As mentioned above, VOCs are secreted by endophytic fungi. In fact, these microorganisms are able to interact with their host plants and produce a mixture of VOCs molecules. The mixture constitutes “sentences” that allow plants to transmit complex signals and, thus, communicate with their environment. The main functions that can be performed by VOCs emitted by plants or fungi are defense against herbivores and pathogens, communication between different organs of the plant or with other plants, and attraction of beneficial agents (for example pollinators, seed dispersers, and microorganisms) [15,21,22]. Therefore, this review is only focused on the current state of knowledge on endophytic fungal VOCs with regard to their potential bioactivity for use against fungal plant pathogens.

## 3. Antifungal Effect of Volatile Organic Compounds Produced by Endophytic Fungi

Several studies highlight the important antifungal role of VOCs compounds. These molecules could have different chemical structures and play a potential role in plant defense against fungal diseases. In their report, Lee et al. [23] demonstrated that, during in vitro and in vivo assays, an antifungal volatile compound produced by *Oxyporus latemarginatus* was found to have inhibitory action against a broad spectrum of pathogenic fungi, including *Alternaria alternata*, *Colletotrichum gloeosporioides*, and *Fusarium oxysporum f. sp. Lycopesici*. The antifungal compound was identified through Gas Chromatography-Mass Spectrometry (GC-MS) as 5-pentyl-2-furaldehyde [23]. Moreover, *O. latemarginatus* EF069 was capable of inhibiting the growth of *Botrytis cinerea* and *Rhizoctonia solani*, which can cause post-harvest apple decay and root rot of moth orchids, respectively. Similarly, Lee et al. [23] revealed that 50 g of wheat bran/rice inoculated with *O. latemarginatus* EF069 effectively reduced the development of post-harvest apple rot caused by *B. cinerea.* In their study, Malmeirca et al. [24] established direct confrontation assays between *Trichoderma* strains and the pathogen *B. cinerea*. For this purpose, agar plug cuts of each fungi were placed in potato dextrose agar plates and incubated at 28 °C in the dark. The radial growth of *Trichoderma. arundinaceum* was observed until it surrounded the pathogen colony. Results revealed that the growth of *B. cinerea* was controlled by *T. arundinaceum*, using trichodiene. This indicates VOCs indirectly inhibits *B.cinerea* by inducing the expression of defense genes encoding the production of salicylic and jasmonic acids and by interacting with hydrolytic enzymes [24]. *Trichoderma sp*. have also been widely used as biological control agents for the control of soil-borne pathogens. VOCs emitted by *Trichoderma harzianum* resulted in 67% growth inhibition of *Colletotrichum capsici,* whereas *Trichoderma saturnisporum* and *Trichoderma reesei* inhibited 59.3% and 30.4% of *C. capsici,* respectively [25]. In the same context, *F. oxysporum* can inhibit the growth of *B. cinerea*. It is also responsible for the total inhibition of three fungal species, *Rhizoctonia solani, Penicillium digitatum,* and *Aspergillus niger*. This has been confirmed by inhibition of pectin methylesterase, cellulase, and polyphenols oxidase secretions [26]. This antifungal activity could be attributed to terpenes, principally limonene, β-phellondrene, and 1,8-cineole (synonym: eucalyptol) secreted by the microorganism. In addition to terpene VOCs secretion, endophytic fungi can emit alcohols and carboxylic acids. For example, *Phomopsis* sp. produce a unique blend of VOCs, which is composed of sabinene, 1-butanol, phenethyl alcohol, 1-propanol, and acetone. This fungus has been isolated as an endophyte associated with *Odontoglossum sp.* (Orchidaceae) in a forest in northern Ecuador. It has been shown that these molecules inhibit a broad spectrum of fungi from different taxonomic groups, such as *Deuteromycetes*, *Ascomycetes*, and *Oomycetes*.

This section emphasizes the description of VOCs compounds secreted by two important endophytic fungi species, *Muscodor* and *Noduliosporium*. *Muscodor* has been the focus of many studies since it produces VOCs known for having lethal effects against a wide variety of pathogenic fungi. Currently, the mixture of VOCs, produced by *Muscodor albus* is used as a primary screening tool to discover new *Muscodor* species with potent VOCs production. To date, 14 *Muscodor* species have been described: *Muscodor albus*, *Muscodor roseus* [27,28], *Muscodor vitigenus* [29], *Muscodor crispans* [30], *Muscodor yucatanensis* [31], *Muscodor fengyangensis* [32], *Muscodor cinnanomi* [33], *Muscodor sutura* [34], *Muscodor musae*, *Muscodor oryzae*, *Muscodor equiseti*, *Muscodor suthepensis* [35], and recently *Muscodor kashayum* [36] and *Muscodor tigerii* [37]. *Muscodor* species have been isolated in South America, the USA, and southeast Asian countries, including Thailand, China, and, most recently, India. The genus *Muscodor* effectively controls several pathogenic fungi. *M. albus* inhibits the growth of *Rhizoctonia solani* and *Phytophthora capsici* responsible for pepper root rot. These species also inhibit the germination of teliospores *Tilletia horrida, Tilletia indica,* and *Tilletia tritici*. Mercier et al. [38] showed that the VOCs spectrum emitted by two strains, *Muscudor* CZ-620 and strain MFC2, effectively controlled fungal rot of multiple fruits. The VOCs mixture was analyzed by GC with Flame Ionization Detector and results showed that it was mainly composed of isobutyl alcohol, 2-methyl-1-butanol, and isobutyric acid [38]. Other *Muscodor* species have been the subject of several studies. *Muscodor cinnamomi* CMU-Cib461 inhibited the growth of *Rhizoctonia solani,* which could induce leaf blight, leaf spot, damping-off, and leaf rot [39]. In the same context, *M. kashayum* inhibited the growth of *Cercosporabeticola*, *Colletotrichum gloesporioides, Mycosphaerella fijiensis, Chaetomium heterosperum,* and *Fusarium oxysporum.* The analysis of volatiles compounds emitted by *M. kashayum* was carried out by a solid phase microextraction coupled with GC-MS. For this purpose, a solid phase microextraction syringe was used to trap the volatile compounds emitted by a 10-day-old culture of *Muscodor* following the method of Ezra et al. [36,40]. Subsequently, VOCs were analyzed by GC-MS. Results revealed that the mixture contains 23 volatile compounds which were identified by comparing the GC-MS spectra. The most abundant of all the volatile compounds produced were cyclohex-3-en-1-ol and β-bisabolol.

These volatiles produced by *M. kashayum* are unique, and have not been previously reported by any other *Muscodor* species, which predominantly produce esters of isobutyric acid, methyl acetate, ethyl-2-methylbutyrate, and alcohol [34,36].

To highlight the antifungal potential of VOCs emitted by endophytic fungi, Strobel et al. [41] introduced the concept of mycofumigation. Mycofumigation is a new biological control alternative for post-harvest diseases of fruit and vegetable rots. However, its effectiveness depends on the fungal species, the amount of inoculum used, and the type of post-harvest disease. Actually, this method is used for the treatment of fruits in storage rather than controlling soil borne pathogens. In this context, soils are inoculated with a *M. albus* preparation in order to preclude the growth of pathogens [17,42]. Field bioassays for the evaluation of *Muscodor albus* efficacy against three pathogenic fungi of sugar beets, *Rhizoctonia solani, Pythium ultimum,* and *Aphanomyces cochlioides,* have been established. Briefly, sterilized barley seeds were inoculated with *M. albus*. After incubation at 25 °C for three weeks, the inoculated grains of barley were dried and ground to a sawdust texture. After that, the preparation was spread over the surface of the soil and covered with black plastic mulch and loose soil. After one week, the soil preparation containing *Muscodor* was added as a top layer onto pots inoculated with pathogens. Sugar beet seeds were planted in different plots and placed in a greenhouse. The amount of healthy sugar beet seedlings was estimated after 14, 21, and 28 days of plantation. Results revealed that mycofumigation of infested soil with *M. albus* improved the healthy seedling establishment of sugar beets [17]. On the basis of several reports highlighting the potential antifungal activity of *Muscodor*, the industrial company AgraQuest, of Davis, CA, USA, is currently undertaking full-scale development of *M*. *albus* for several agricultural applications [43]. Different aspects related to the optimization of the formulation, cost-effectiveness ratio, and scale-up of mycofumigation with Muscodor are underway. This may limit the utilization of others hazardous fumigants such as methyl bromide chloropicrin mixtures [44,45]. In their study, Suwannarach et al. [35] established an in vivo assay for investigating fumigation activity of *M. suthepensis.* Briefly, the sterilized surface of tangerine fruits was inoculated with spore suspensions of the pathogenic fungi *P. digitatum.* Next, the infected fruits were stored at 25 °C in plastic boxes containing inoculum of *M. suthepensis.* After 24 h, the fungal inoculum was removed, and boxes were kept at 25 °C for nine days. The diameters of decay lesions were measured during the incubation period. Results showed that a 12 h fumigation with 30 g per 4 L of a *M. suthepensis* inoculum completely controlled mandarin fruit rot caused by the pathogenic fungi *P. digitatum* [39]. Similarly, a 24 h fumigation with 30 g per 11.4 L of rye grains of *M. albus* was required for the complete control of *P. digitatum*. Moreover, 24 h of fumigation with 30 g per 11.4 L of *M. albus* inoculum controlled blue mold caused by *Penicillium expansum* and gray mold caused by *B. cinerea* of apples. Fumigation with 140 g per 11.4 L of *M. albus* inoculum controlled brown rot caused by *Monilinia fructicola*. In their report, Saxena et al. [37] revealed that growth of *Alternaria alternans* and *Cercospora beticola* were totally inhibited by volatile compounds secreted by *Muscodor tigerii*. In other studies, it has been demonstrated that the VOCS mixture produced by *Muscodor CZ-620* and *MFC2* limited fungi rot in a wide range of fruits. Recently, Hutchings et al. [46] identified a novel VOC molecule, N-methyl-N-nitrosoisobutyramide (MNIBA). This thermolabile molecule was identified by GC-MS analysis when a low-injection temperature (140 °C) was applied. It has been reported that the toxicity of *M. albus* is directly correlated to the MNIBA concentration in the VOCs mixture produced by this fungal strain. MNIBA is known to cause DNA damage by methylation. The spontaneous decomposition of MNIBA compounds generates, principally, iso-butyric acid and methyl-diazohydroxide, which could be converted, in a second step, to methyl-diazonium. This compound induces DNA methylation and, therefore, the generation of NO radicals, which are responsible for the chemical nitrosylation of amides. GC-MS analysis of COVs produced by *Muscodor* revealed a high amount of a volatile compound derived from isobutyric acid. The higher bioactivity of MNIBA is attributed to the presence of this compound. Hence, *Muscodor* species are considered as potential agents that could be used in biofumigation.

*Nodulisporium* sp. are characterized by their resistance to VOCs emitted by *M. albus.* This resistance is assigned to the ability of these fungi to produce VOCs with important antifungal properties [47]. Suwannarach et al. [48] showed that the strain *Nodulisporium* sp. CMU-UPE34 was able to produce 31 VOCs. Analyses of the VOCs mixture by GC-MS revealed that it consisted mainly of alcohols, acids, esters, and monoterpenes. Among these molecules, eucalyptol (synonym: 1,8-cineole) was the most abundant volatile compound. In the second part of this work, an in vivo assay for fumigation activity of volatile compounds produced by *Nodulisporium* was carried out. For this purpose, sterilized surfaces of citrus fruits were inoculated with different pathogenic fungi and incubated in plastic boxes. Next, boxes were inoculated with *Nodulisporium* sp. CMU-UPE34. After 48h of incubation at 25 °C, the fungal inoculum was removed, and the boxes were re-stored in the same conditions. After one week, the diameters of decay lesions by each pathogen were measured. Results revealed that the mixture of VOCs inhibited the growth of different pathogenic fungi with the inhibition yield ranging from 47%–93% [48]. In addition, in vivo tests have shown that *Nodulisporium sp.* CMU-UPE34 controlled the growth of *Penicillium digitatum* and *Penicillium expansum* and caused completely inhibition of *Aspergillus fumigatus* and *Rhizoctonia solani* [49]. Other studies demonstrated that the strain *Nodulisporium* sp. CMU-UPE34 efficiently inhibited the proliferation of *P. digitatum*, *P. expansum*, *Aspergillus fumigatus*, and *R. solani* [50]. Analysis of the VOCS mixture secreted by this fungal strain revealed that it was mainly composed of 1,8-cineole and terpinen-4-ol. The compound 1,8-cineole can cross cellular membranes and induce damage in different cellular organelles. Moreover, it has been shown that there is a synergistic effect between 1,8-cineole and terpinen-4-ol. In fact, 1,8-cineole can easily cross the cellular membrane and, therefore, facilitate the entry of terpinen-4-ol into the intracellular medium [51]. Other studies have reported that the fumigation of jasmine rice, which is a variety of *Oryzaindica,* with 50 g and 60 g of *Nodulisporium* sp. CMU-UPE34 culture and the fumigation of wheat/rice bran with 50 g of *Nodulisporium sp.* CF016 controls cabbage green mold completely and suppresses the development of gray and blue mold lesions by 88% and 76%, respectively [39,52,53].

To better understand the antifungal effect of VOCs on pathogenic fungi, researchers have used artificial molecules. They compared them with VOCs secreted directly from fungi. According to the study established by Syed et al. [54], in comparison with fungal VOCs, a mixture of synthetic VOCs consisting of pentan-2-one, hexan-3-one, 1,8-cineole, β-farnesene, and propanoic acid was more effective against *Phytophthora palmivora*, *Phytophthor cinnamomi*, *Pythium ultimum*, *R. solani*, *Sclerotinia sclerotiorum,* and *B. cinerea* [54]. In addition, a comparative study between the natural thujospen emitted by *Penicillium decumbens* Thom C. and commercial thujospen showed that they had similar antifungal bioactivity against the five strains tested—*Aspergillus sydowii*, *Eurotium herbariorum*, *Polytrichum macroclada*, *Penicillium hirsutum*, and *P. decumbens* [55]. In addition, Singh et al. [56] showed that the use of the median effective concentration (EC50) of an artificial mixture similar to the VOCs emitted by *Phomopsis* sp. completely inhibited the growth of *Colletotrichum lagenarium* and *Trichoderma viride* [56]. However, natural VOCs showed no inhibitory effect on *T. viride* and *C. lagenarium*. This suggests that some VOCs emitted by *Phomopsis* sp. but not identified through GC-MS due to low concentrations that do not reach the detection threshold, may influence the inhibitory effect on the fungus [56]. In the same context, the use of a pure chemical or a mixture of several chemicals (butyl, ethyl acetate, and ethanol representing the VOCs spectrum naturally emitted by *Ceratocystis fimbriata*), in proportions calculated using GC-MS analysis, showed no inhibitory effect. According to the authors, the inhibition may be a synergistic effect of all VOCs of *C. fimbriata*, including molecules not detected using current identification methods [57]. A similar observation was recorded for another VOCs mixture produced by *Muscodor* sp.

## 4. Phytoxicity of Volatile Organic Compounds

Despite all the promising results, the question of phytotoxicity depended on the use of VOCs. Numerous studies have shown strong relationships between VOCs and phytotoxicity [58]. As early as 1965, Muller confirmed that terpenic VOCs of *Sarracenia leucophylla* reduced the number of rootlets and the germination of hypocotyl seeds of *Cucumis sativus (L.)* [59]. Inderjit et al. [60] revealed that species richness of plants was much lower due to VOCs produced by *Ageratina adenophora* litter. In the same context, He et al. [61] showed that dead leaf water and decomposition of residues around *Eucalyptus urophylla* contained VOCs of the sesquiterpenes 1,8-cineole and terpinene-4-ol, and, respectively, represented 19% and 39% of the water-soluble mixture. The synthetic forms of these two volatile compounds inhibited the germination of some cereals, and the growth of some weeds [61]. Lee et al. [62] studied the phytotoxicity of alcoholic VOCs. Exposure of *Arabidopsis thaliana (L.)* grains and plants to a concentration of 1 PPM 1-octanol, 2-octanol, 3-octanol, and 1-octen-3-ol synthetic forms showed no effect on germination. Nevertheless, these molecules caused a growth retardation of the radical part. In the same study, different plant and ethanol samples were examined, as well as their morphological changes or modifications. The chlorophyll concentration and root growth of treated plants was also measured. These results are confirmed by Ogura et al. [63] who tested the phytotoxicity of alcohols emitted by a non-endophytic fungi *P. expansum*. The results were analyzed with a concentration of 100 mg/L, completely inhibiting the germination of 15 types of Brassicaceae, including six radish varieties. This information could also be important when considering the phytotoxic effect of VOCs, including their dose, origin, chemical nature, and mode of application. Table 1 summarizes proprieties of the most important endophytic fungi VOCs.

## 5. Conclusions

This review summarizes literature regarding the bioactivity of volatile substances produced by endophytic fungi. There is a consensus that VOCs emitted by endophytic fungi lead to partial or total inhibition of pathogenic fungi growth. To this end, the emission of antifungal VOCs appears to be a promising way to limit the use of pesticides for controlling fungal plant pathogens. However, it is important to mention that the antimicrobial activity of VOCs depends closely on their origin, dose, and application form. Currently, the great bioactivity potential of these compounds is drawing the attention of industry to commercialize VOC products for agricultural applications. However, multiple efforts need to be launched for the industrial production of theses formulations. Fungal VOCs are emitted in small quantities which could prevent their commercialization. In order to promote VOCs products, more studies must focus on determining the appropriate methods to ensure the greatest cost-effectiveness ratio. Ultimately, by using genetic tools, key genes involved in VOCs biosynthetic pathways can be identified and over-expressed for large-scale production of these compounds. Since a number of reports highlighted the phytotoxicty of some VOCs, more studies need to be conducted to safely use these molecules for biofumigation. Moreover, the toxicity of VOCs for humans needs to be strictly evaluated before their use in biocontrol strategies.

As endophytic fungi are abundant and have high genetic diversity, the identification and characterization of novel VOCs is a current research topic.

## Figures and Tables

**Table 1:** Overview of VOCs molecules, their origins, and antimicrobial effects.

Endophytic Fungi Producing VOCs	VOCs Effects	Microorganisms Inhibited by VOCs	VOCs/CAS Number	Molecular Formula	Hosts	References
Fusarium oxysporum can-* 46	Inhibition of mycelium	*Aspergillus flavus* *Aspergillus parasiticus* *Botrytis cinerea* *Colletotrichum siamense* *Fusarium graminearum* *Fusarium oxysporum f.sp. vasinfectum* *Magnaporthe oryzae* *Monilinia fructicola*	Farnesol (4602-84-0) β-caryophyllene (87-44-5) Limonene (138-86-3) Hexanoic acid (142-62-1) Octanoic acid (124-07-2)	C_15_H_26_O C_15_H_24_ C_10_H_16_ C_6_H_12_O_2_ C_8_H_16_O_2_	*Gossypium herbaceum*	[64]
*Hypoxylon sp.*	Inhibition of mycelium	*Botrytis cinerea* *Ceratocystis ulmi* *Cercospora beticola* *Colletotrichum lagenarium* *Fusarium solani* *Geotrichum candidum* *Mycosphaerella fijiensis* *Phytophthor apalmivora* *Phytophthor acinnamomi* *Rhizoctonia solani* *Sclerotinia sclerotiorum* *Trichoderma viride* *Verticillium dahliae*	1,8-cineole (470-82-6) 1-methyl-1,4-cyclohexadiene (4313-57-9)	C_10_H_18_O C_7_H_10_	*Persea indica*	[65]
*Muscodor albus*	Complete inhibition of mycelium growth	*Pythium ultimum* *Rhizoctonia solani* *Tapesia yallundae*	Methylacetate (79-20-9)	C_3_H_6_O_2_	*Cinnamomum zeylanicum*	[39]
	Inhibition of mycelium	*Botrytis cinerea* *Monilinia fructicola* *Penicillium expansum*	2-methyl-1-butanol (137-32-6) 2-methylpropionic acid (79-31-2)	C_5_H_12_O C_4_H_8_O		[66]
	Inhibition of mycelium	*Aspergillus ochraceus* *Fusarium solani* *Rhizoctonia solani* *Sclerotinia sclerotiorum*	2-methylpropionic acid (79-31-2)	C_4_H_8_O_2_		[67]
	Inhibits the germination of teliospores	*Tilletia horrida* *Tilletia indica* *Tilletia tritici*	Unknown			[68]
	Inhibition of mycelium growth	*Helminthosporium solani* *Fusarium sambucinum*	2-methylpropionic acid (79-31-2) 3-methyl-1-butanol (123-51-3) Ethyl alcohol (64-17-5)	C_4_H_8_O_2_ C_5_H_12_O C_2_H_6_O		[69]
	Complete inhibition of mycelium growth	*Aspergillus sp* *Colletotrichum sp* *Geotrichum sp.*	N-methyl-N-nitrosoisobutyramide (1255641-06-5)	C_5_H_10_N_2_O_2_		[46]
*Muscodor musae*	Inhibition of mycelium	*Alternaria porri* *Alternaria solani* *Aspergillus flavus* *Botrytis cinerea* *Colletotrichum capsic* *Colletotrichum gloeosporioides* *Colletotrichum musae* *Fusarium oxysporum* *Fusarium solani* *Nigrospora oryzae* *Penicillium digitatum* *Penicillium expansum* *Rhizoctonia solani* *Sclerotium rolfsii* *Candida albicans* *Cryptococcus neoformans* *Escherichia coli* *Enterococcus faecalis* *Proteus mirabilis* *Staphylococcus aureus* *Streptococcus pneumoniae*	Isobutyric acid (79-31-2) 3-methyl-1-butanol (123-51-3) Ethyl-2-methylbutyrate (7452-79-1)	C_4_H_8_O_2_	*Musa acuminata*	[35]
*Muscodor equiseti*				C_5_H_12_O C_7_H_14_O_2_	*Equisetum debile*	
*Muscodor oryzae*					*Oryza rufipogon*	
*Muscodor cinnamomi*					*C. bejolghota*	
*Muscodor suthepensis*					*C. bejolghota*	
*Muscodor Darjeelingensis*	Inhibition of mycelium	*Alternaria alternata* *Arthrinium phaeospermum* *Aspergillus flavus* *Aspergillus niger* *Bionectria ochroleuca* *Botrytis cinerea MTCC 359* *Cercospora beticola* *Colletotrichum gloeosporioides MTCC 9623* *Fusarium solani* *Fusarium oxysporum* *Lasiodiplodia theobromae* *Muscodor albu scz620* *Penicillium chrysogenum* *Rhizoctonia solani* *Talaromyces marneffei* *Candida glabrata* *Candida viswanathii* *Pseudomonas aeruginosa MTCC 3541* *Pseudomonas aeruginosa MTCC 647r* *Staphylococcus epidermidis MTCC 2639*	Isobutyric acid (79-31-2) 3-methyl-1-butanol (123-51-3) Ethyl-2-methyl-butyrate (7452-79-1)	C_4_H_8_O_2_ C_5_H_12_O C_7_H_14_O_2_	*C. camphora*	[70]
*Muscodor kashyum*	Inhibition of mycelium growth	*Alternaria alternata MTCC5432* *Agaricus bisporus* *Aspergillus japonicus* *Bionectria ochroleuca* *Candida albicans* *Cercospora beticola* *Chaetomium heterosporum* *Colletrotrichum gloeosporioides* *Curvularia lunata* *Fusarium equiseti* *Fusarium oxysporum* *Lasiodiplodia theobromae* *Muscodor albus CZ620* *Mycosphaerella fijiensis* *Penicillium citreonigrum* *Penicillium marneffei* *Trichoderma viride* *Pleurotus flabellatus*	cyclohex-3-en-1-ol (822-66-2) β-Bisabolol (15352-77-9)	C_6_H_10_O C_15_H_26_O	*Aegle marmelos*	[36]
*Nodulisporium sp.*	Inhibition of mycelium	*Aspergillus fumigatus* *Aspergillus flavus* *Botrytis cinerea* *Colletotrichum lagenarium* *Ceratocystis ulmi* *Cercospora beticola* *Fusarium solani* *Geotrichumcandidum* *Phytophthor apalmivora* *Phytophthora cinnamoni* *Pythium ultimum* *Rhizoctonia solani* *Sclerotinias clerotiorum* *Trichoderma viridae* *Verticillium dahlia*	1-methyl-1,4-cyclohexadiene (4313-57-9) 2-methyl-1-pentanol (105-30-6) 1-Heptanol (111-70-6) 1-Octanol (111-87-5)	C_7_H_10_ C_6_H_14_O C_7_H_16_O C_8_H_18_O		[54]
*Nodulisporium* sp. *CMU-UPE34*	Inhibition of mycelium growth	*Alternaria porri* *Alternaria solani* *Colletotrichum capsici* *Colletotrichum musae* *Colletotrichum gloeosporioides* *Fusarium oxysporum* *Penicillium digitatum* *Penicillium expansum* *Nigrospora oryzae* *Rhizoctonia solani* *Sclerotium rolfsii*	1,2,4-trimethylenecyclo-hexane (2234-75-5) 3-methyl-1-butanol (123-51-3) Limonene (138-86-3) Eucalyptol (synonym: 1,8-cineole) (470-82-6) β-myrcene (123-35-3) Terpinen-4-ol (562-74-3)	C_9_H_18_ C_5_H_12_O C_10_H_16_ C_10_H_18_O C_10_H_16_ C_10_H_18_O		[48]
*Oxyporus latem arginatus EF069*	Inhibition of mycelium growth	*Alternaria alternata* *Botrytis cinerea* *Colletotrichum gloeosporioides* *Fusarium oxysporum f.sp. lycopersici*	2-Furanmethanol (90200-14-9)	C_8_H_12_O_4_	*Capsicum annum*	[23]
*Phomopsis sp.*	Inhibition of mycelium growth	*Aspergillus fumigatus* *Ceratocystis ulmi* *Colletotrichum lagenarium* *Geotrichum candidum* *Phytophthora palmivora* *Pythium ultimum* *Rhizoctonia solani* *Sclerotinia sclerotiorum*	Sabinene (3387-41-5) 3-methy-1-butanol (123-51-3) 1-Propanol (71-23-8) 2-Propanone (67-64-1)	C_10_H_16_ C_5_H_12_O C_3_H_8_O C_3_H_6_O	*Odontoglossum sp.*	[56]
*Trichoderma harzianum T-E5*	Inhibition of mycelium growth	*Fusarium oxysporum f.sp. cucumerinum (FOC)*	Diterpene (146985-82-2)	C_20_H_30_O_4_	Cucumis sativus *	[26,71]
*Trichoderma saturnisporum*	Inhibition of mycelium growth	*Colletotrichum capsici*	Ethylene (74-85-1) Hydrogen cyanide (74-90-8)	C_2_H_4_ CHN	Capsicum frutescence) *	[25]
*Trichoderma reesei*					Capsicum frutescence *	
*Trichoderma* *harzianum*					Capsicum frutescence *	
*Trichoderma viride*	Inhibition of mycelium growth	*Botrytis cinerea* *Fusarium oxysporum*	6-pentyl-2H-Pyran-2-one (27593-23-3)	C_10_H_14_O_2_	Capsicum frutescence *	[72]
*Trichoderma harzianum*	Induces the expression of tomato defense genes related to salicylic acid (SA)	*Botrytis cinerea*	Diterpene (146985-82-2)	C_20_H_30_O_4_	Solanum lycopersicum *	[73]

* *Fusarium oxysporum* and *Trichoderma* sp. were found in various plant. Examples of theses hosts are presented in the table.
